# Allocation of Oral Cholera Vaccines in Africa

**DOI:** 10.3390/vaccines13050519

**Published:** 2025-05-14

**Authors:** Elisa M. Maffioli, Yutong Lu

**Affiliations:** 1 Department of Health Management and Policy, School of Public Health, University of Michigan, Ann Arbor, MI 48109, USA; 2 Department of Global Health and Population, Harvard T.H. Chan School of Public Health, Boston, MA 02115, USA; luyutong04@gmail.com

**Keywords:** cholera, disease outbreak, vaccines, resource allocation

## Abstract

Objectives: In this study, we examine the allocation of oral cholera vaccines (OCVs) across 25 African countries between 2013 and 2019. Methods: We constructed a dataset combining cholera outbreaks and requests, decisions, and deliveries of OCVs from the Global Task Force on Cholera Control, alongside additional covariates. Using machine learning algorithms, we assess the relative importance of socio-demographic, governance, and weather variables in predicting cholera outbreaks. We constructed and used an “index of cholera risk” as an instrumental variable to predict the likelihood of suspected cases and estimate the impact of cholera outbreaks on OCV allocation. Results: The majority of OCVs (77.4%) were allocated reactively. Governments took an average of 299.6 days to request doses, international agencies took 10.4 days to decide, and it took 84 days for vaccines to be delivered. Countries experiencing a cholera outbreak were 31.7 and 36.5 percentage points more likely to request and receive a vaccine delivery in the same month as the outbreak, respectively. We confirmed that the probability of obtaining vaccines through a reactive mechanism was 48.4 percentage points higher compared to preventive allocation. When exploring the heterogeneity of impacts, OCVs were more likely to be requested, allocated, and delivered in countries with strong institutions and those not facing crisis situations. OCVs were also more likely to be allocated in the central parts of the continent. Conclusions: While OCV allocation is responsive to cholera outbreaks, addressing delays, particularly in high-risk countries, could improve their distribution and mitigate the impact of cholera outbreaks. This study highlights the need for targeted strategies to ensure vaccine access in fragile and conflict-affected settings, where institutional capacity is weaker.

## 1. Introduction

Cholera is an acute diarrheal disease caused by ingesting food or water contaminated with the bacterium *Vibrio cholerae*. Despite being preventable and treatable, cholera remains a significant public health challenge, particularly in countries with inadequate access to safe water and improved sanitation. The World Health Organization (WHO) estimates that cholera causes between 1.3 and 4 million cases and results in 21,000 to 143,000 deaths annually [1]. Cholera is endemic in many regions of Asia and Africa, with the highest risk affecting populations living without access to safe water, sanitation, and adequate hygiene [2]. Sub-Saharan Africa is among the hardest-hit regions, with over 230,000 cases and 4000 deaths reported across 14 countries since the start of 2023 [3].

The resurgence of cholera in Africa has been increasingly linked to the impacts of climate change [4,5]. Rising temperatures, changing rainfall patterns, and extreme weather events create conditions that facilitate the spread of *Vibrio cholerae*. For example, flooding can contaminate water sources with sewage, while droughts limit access to safe drinking water, forcing reliance on unsafe alternatives. In coastal areas, warming sea temperatures and changing ecosystems promote cholera proliferation. Climate-related displacement and strained sanitation infrastructure in densely populated regions further increase vulnerability to outbreaks. As climate change intensifies, the risk of cholera is expected to rise, especially in sub-Saharan Africa, where fragile health systems are ill-equipped to manage this threat.

The oral cholera vaccine (OCV) is an effective tool for preventing cholera outbreaks. Administered orally in two doses, the first followed by a second dose within 1 to 6 weeks, OCV provides protection for up to five years, although its effectiveness is lower in children under five [6]. However, the supply of OCV is limited [7]. In 2018, 23 million doses were produced, sufficient for 11.5 million people, which is just a fraction of the 87.2 million people living in high-risk areas of sub-Saharan Africa [8]. As of September 2024, 33 countries have requested a total of 352.8 million doses, but only 184.5 million doses were delivered [9]. After the global stockpile was depleted in October 2024, the production ramped up in November with new formulations and production methods introduced and prequalified [10,11]. Yet, the ongoing scarcity continues to obstruct efforts to manage cholera outbreaks and to respond swiftly to the disease’s spread.

Given the limited global supply, countries do not routinely administer the OCV; instead, it is primarily used reactively during emergency outbreaks. In public health emergencies, a single dose is often administered to provide short-term protection. This approach allows for quicker coverage of large groups, which is crucial in emergency settings like refugee camps or during natural disasters. However, this one-dose strategy typically offers protection for only about six months, compared to the longer-lasting immunity provided by two doses [12]. After the immediate threat is managed, it is recommended to administer a second dose to ensure sustained immunity, especially in endemic regions.

The OCV allocation in Africa is supported by global partners such as the WHO and the Global Alliance for Vaccines and Immunization (GAVI), regional organizations, and national governments working together to address the public health challenge of cholera, with a focus on integrating vaccination with broader water, sanitation, and hygiene (WASH) interventions. The WHO, particularly through the Global Task Force on Cholera Control (GTFCC), provides a strategic framework and technical support to ensure efficient vaccine distribution in high-risk areas. GAVI provides funding and supports countries in vaccine procurement, working to ensure that OCVs are affordable and accessible to vulnerable populations in Africa.

There exists an emergency stockpile of vaccines, funded by GAVI, the WHO, and other partners, which is managed by the International Coordinating Group (ICG). To request access to OCV, a country needs to submit an OCV request form, annexes and other required documents to the ICG secretariat. An ICG member agency present in the country (WHO, Médecins Sans Frontières (MSF), the International Federation of Red Cross and Red Crescent Societies (IFRC) and the United Nations International Children’s Emergency Fund (UNICEF)) can also submit the application on behalf of the Ministry of Health (MOH). The request form to access ICG support from the global stockpile includes (i) epidemiological information per place and week (cases, deaths, attack rate, case fatality rate); (ii) history of previous outbreaks of cholera in the last 3 years (year and month, duration, cases and deaths, any vaccination, doses); (iii) laboratory information as 10–20 cases need to be confirmed to ascertain a cholera outbreak; and (iv) estimate of vaccine needs (area, population, urban vs. rural, and expected number of doses), in addition to a vaccination plan and a map of areas to be vaccinated. In addition, the request form asks for basic information on capacity to control the outbreak such as availability of facilities for patient care, WASH interventions, behavioral and social interventions, and cold chain capacity. Requests are then evaluated based on the severity of the outbreak, the population at risk, and the country’s capacity to implement a vaccination campaign. Once approved, vaccines are dispatched to the country experiencing an outbreak. These emergency vaccinations are often complemented by other WASH interventions to prevent further spread. For example, countries like Mozambique, Malawi, and South Sudan have received large shipments during outbreaks exacerbated by natural disasters.

Another mechanism of allocation which GAVI primarily supports is for preventive campaigns in endemic areas. For example, in endemic areas, such as Ethiopia, Nigeria, and Sudan, vaccines are distributed to vulnerable populations with poor sanitation and limited access to clean water to prevent outbreaks before they occur, as conflict and displacement have increased cholera vulnerability. This decision is based on data from cholera surveillance systems, which assess the historical burden of cholera, population density, access to clean water, and healthcare infrastructure [9]. In addition, countries develop national cholera control plans in collaboration with the GTFCC, which outline target populations, prioritize high-risk areas (e.g., refugee camps, urban slums, or regions with poor sanitation), and coordinate efforts among ministries of health, WHO, and partners. National health systems, along with non-governmental organizations help with the last-mile delivery of vaccines, ensuring they reach rural or hard-to-access communities [5,9].

In May 2018, the 71st World Health Assembly adopted a resolution to reduce global cholera deaths by 90% by 2030 [13]. Achieving significant reductions in morbidity and mortality is essential to this goal, especially in sub-Saharan Africa. The allocation of OCVs in Africa is then crucial for controlling both endemic and epidemic cholera, with vaccination campaigns often combined with long-term improvements in water and sanitation for sustainable prevention. Consequently, efficient strategies are essential to maximize the impact of the limited vaccine supply in cholera control. However, there remains limited understanding of how the allocation process functions in practice and the criteria considered. There is limited evidence on how OCVs are allocated in the real world, while evidence on optimal allocation is primarily confined to modeling studies [14,15]. Moore et al. (2015) modeled the allocation of OCVs in endemic regions and found that the best strategy is to allocate most doses to reactive campaigns unless the request came late in the epidemic, while unused doses should be distributed in proactive campaigns [14]. Lee et al. (2019) projected impacts of OCVs in sub-Saharan Africa and found that an approach optimized to targeting regions by historical burden rather than risk factors such as limited access to water and sanitation would be more cost-effective in terms of cost per disability-adjusted life years (DALYs) [15].

This paper aims to investigate the allocation of OCVs across African countries. By evaluating the relative importance of socio-demographic, governance, and weather variables in predicting cholera outbreaks, we develop an “index of cholera risk” at the country–month level. This index serves as an instrumental variable to predict the likelihood of suspected cholera cases and assess the impact of cholera outbreaks on OCV allocation. Furthermore, we analyze heterogeneous impacts to identify gaps in vaccine distribution, with the aim of improving proactive planning and strengthening governance mechanisms to ensure equitable and efficient OCV allocation across the continent.

## 2. Material and Methods

### 2.1. Data

We used data on cholera outbreaks [16] and data from the GTFCC dashboard [9] to investigate how OCVs were allocated across 25 countries in Africa between 2013 and 2019. For the analysis, we selected African countries that either had an outbreak recorded or a request, decision or delivery of OCVs in the datasets over the study period (Table A1 for a list of countries).

In terms of cholera outbreak characteristics, the data [16] contained information on any or number of (suspected, confirmed, death) cases, duration of the outbreak (weeks), time to peak of the outbreak (the number of weeks between the start of an outbreak and the week with the most reported cases), attack rate, threshold, and case fatality rate. Based on these data, we also constructed the total number of (confirmed) cases over the previous 3 years.

In terms of allocation of OCVs, the GTFCC data contained any and number of requests, decisions and deliveries, and the associated doses, together with the number of days taken to decide and deliver OCVs. After combining the outbreak and the OCV datasets, we also constructed the numbers taken from the last outbreak to the first request.

Finally, we constructed a dataset of covariates to further account for time variant characteristics for each country. This includes (i) Socio-demographic variables: GDP per capita (in 1000 USD) [17], population [18], population density [19], refugee population [20], net migration [21], share of urban population [22], total number of conflicts and fatal ones [23], and the share of the population with unimproved drinking water [24] and sanitation services [24]. (ii) Governance variables that measure control of corruption, government effectiveness, and political stability and absence of violence/terrorism [25,26], and a manually constructed indicator for whether an election happened [27] to account for possible interruptions in the logistics of requesting or delivering OCVs. (iii) Weather variables include monthly mean precipitation (in millimeters), monthly air temperature between 20 and 25 degrees Celsius) [28], and the number of floods [29]. As far as air temperature is concerned, cholera is found to be correlated with temperature [30]. However, a systematic literature review shows how this relationship is not linear [31]. As Vibrio cholera grows optimally at temperatures between 20 °C and 45 °C [32], we considered different temperature ranges and ultimately selected 20–25 °C as the most predictive for our measure of outbreak risk. Please see Table A2 for a list of all variables considered, and their measurements and links to data sources.

### 2.2. Identification Strategy

Our dataset, which combines data on cholera outbreak and allocation of OCVs and covariates, is at the month–country level (25 countries are observed for 12 months for 7 years, N = 2100). We initially explore the allocation of OCVs in terms of requests, decisions, and deliveries by investigating how the outbreak characteristics are associated with measures of OCVs allocation.

Our empirical model relies on an ordinary least squares (OLS) regression:

(1) $$Y_{cym}=a_{1}+b_{1}{Outbreak}_{cym}+c_{1}X_{cym}+c+ym+e_{cym}$$

where *Y_cym_* refers to the measures of request, decision, and delivery of OCV, including any dose requested, decided on, or delivered; total number of doses by category; number of requests, decisions, and deliveries; total number of days from outbreak to request, from request to first decision, from decision to first delivery; in country *c*; year *y*; and month *m*. *Outbreak_cym_* refers to a proxy for whether there was a cholera outbreak that we primarily measure based on suspected cases, and *X_cym_* is a vector of covariates described above. We also control for year–month (*ym*) and country (*c*) fixed effects, thus exploiting variation within the country over the study period. Standard errors are clustered at the country level. Analyses were performed using Stata/MP, version 18.0 (StataCorp, LLC, College Station, TX, USA).

However, cholera outbreaks can be endogenous when analyzing their impact on vaccine allocation because outbreak incidence might itself influence factors that affect OCVs distribution. Thus, we consider an instrumental variable (IV) approach that could help estimate the causal impact of cholera outbreaks on OCV allocation.

**Figure 1:**
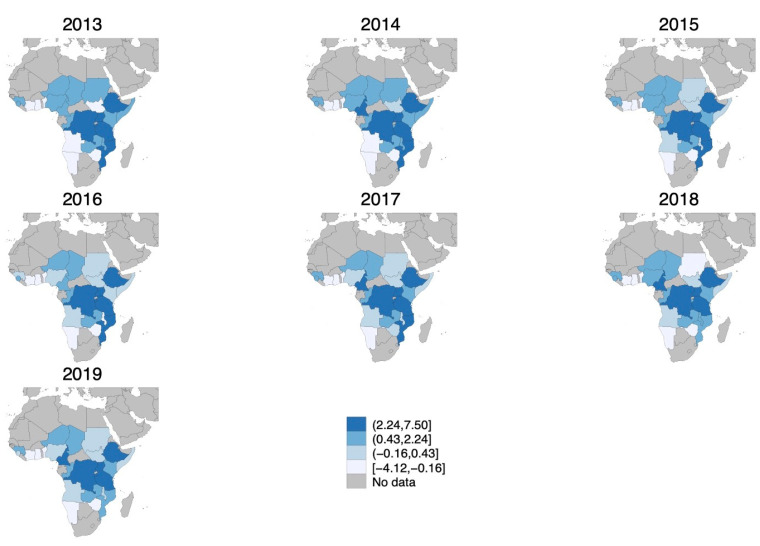
Cholera risk index over study period (in standard deviations). Gray areas represent no data availability. Darker blue shaded areas indicate higher cholera risk index. Cut-offs are fixed and represent the 25th, 50th, and 75th percentiles in 2016. Minimum and maximum values were adjusted to include all values across the years of the study period (2013–2019).

We use least absolute shrinkage and selection operator (LASSO) estimation to assess the relative importance of socio-demographic, governance, and weather variables in predicting cholera outbreaks. The LASSO is a regression analysis method that adds a penalization term based on the sum of the absolute values of the coefficients. By shrinking the parameters toward zero, it enhances the prediction accuracy and the interpretability of the model. The output entails only those variables that are the most relevant predictors [33]. We use all the covariates listed above and defined in Table A2 to predict cholera outbreaks—defined as at least a recorded suspected case—on the sample of countries with at least a cholera outbreak over the study period. The algorithm selects the proportion of population with improved water, with improved sanitation, political stability and absence of violence/terrorism, monthly mean precipitation, and temperature between 20 and 25 degrees Celsius. We use these variables to construct a cholera risk index by standardizing each item following [34] and use it as an IV for whether the country recorded at least a suspected cholera case in that month–year. Figure 1 depicts how the cholera risk index changed over the study period. From 2013 to 2019, cholera risk in Africa shifted from west to east, with the Central and Eastern regions experiencing persistently higher cholera risk, while West Africa generally saw declining outbreaks.

Two conditions need to hold for the cholera risk index to be a valid IV. First, the IV needs to be relevant, i.e., the cholera risk index has to be correlated with the regressor of interest. We implemented the LASSO on several measures of outbreaks following the criteria used to evaluate countries’ requests for OCVs and found that the most valid IV is any suspected cases, followed by mean R0, which we use as robustness check for the results (Table A3). Controlling for time and country fixed effects and clustering standard errors at the country level, we find that 1 standard deviation increase in cholera risk index increases the likelihood of having an outbreak by 2.37 pp, which represents a 23.6% increase over the mean of 10% of any suspected cholera cases recorded in the sample. Cragg–Donald Wald F statistics for IV weak identification test are 21.691 for outcomes at time t, 21.686 for outcomes at time + 1, and 21.624 for outcomes at t + 3, suggesting the IV is not weak.

Second, the instrument must be exogenous: the cholera risk index should be uncorrelated with the error term, meaning it should influence OCV allocation only through its effect on cholera outbreaks. The primary threat to this identification strategy is if the cholera risk index is correlated with unobserved confounders that directly affect OCV allocation. First, if countries rely on factors included in the cholera risk index to allocate vaccines, the exclusion restriction could be violated. However, the key criteria for vaccine allocation—such as epidemiological data, past outbreaks, and laboratory confirmation—are largely absent from the predictors used to construct the index. While some other characteristics (e.g., population, living in urban areas) may be relevant to vaccine needs, they were not selected by the LASSO and thus do not contribute to the index. Additionally, although vaccine requests require reporting on WASH programs, these interventions are not included as predictors. Second, the cholera risk index may correlate with other factors influencing OCV allocation such as donor funding and media attention, which may direct vaccines toward certain countries, independent of cholera risk. A particular concern is the inclusion in the index of the proportion of the population with unimproved drinking water or sanitation services. While exogeneity is untestable, we confirmed that the correlation among these measures and OCV delivery is not statistically significantly, suggesting no direct pathway for OCV allocation unless a cholera outbreak occurs. However, we cannot rule out the possibility that the proportion of the population with unimproved drinking water or sanitation services is correlated with other determinants of OCV allocation, such as international aid priorities, health system capacity, or WASH interventions. If donors target countries with poor water access for broader cholera prevention efforts beyond outbreak response, this may violate the exclusion restriction. Taking these caveats into consideration, our preferred empirical model is a two-stage least square, as follows:

(2) $${Outbreak}_{cym}=a_{2}+b_{2}{Cholera\;Risk\;Index}_{cym}+c_{2}X_{cym}+c+ym+e_{cym}$$

(3) $$Y_{cym}=a_{3}+b_{3}\hat{{Outbreak}_{cym}}+c_{3}X_{cym}+c+ym+e_{cym}$$

where *Cholera Risk Index_cym_* refers to constructed cholera risk index as predicted by the LASSO and used as an IV, while *Outbreak_cym_* and *X_cym_* are defined as above. We control for year–month (*ym*) and country (*c*) fixed effects, and standard errors are clustered at the country level. This methodology helps to mitigate the endogeneity problem by using the cholera risk index, which is correlated with the likelihood of an outbreak but is presumably not directly influencing OCV allocation decisions beyond the outbreak itself. We aim to causally estimate $b_{3}$.

### 2.3. Additional Analysis

Furthermore, we explored additional effects by investigating the agencies that made requests (MOH, WHO, Agence de Médecine Préventive (AMP), MSF, STChealth (STC)) or approved (GTFCC, ICG) the vaccines. We also considered the allocation of different types of OCVs (Euvichol, Euvichol Plus, and Shanchol) and the mechanism of allocation (reactive compared to preventive).

Finally, we constructed measures of country vulnerability—such as weak institutions and crisis situations—to explore heterogeneous effects, which we derived from several pre-existing covariates. Specifically, we generated variables to identify countries with weak institutions, defined as those scoring below the median for government effectiveness and control of corruption. This resulted in 43% of the sample being classified as having weak institutions. Additionally, we defined a crisis variable, identifying countries contending with refugee populations and number of conflicts higher than the median in the sample, resulting in 33% of the sample categorized under crisis conditions. These categorizations allow us to investigate how institutional weaknesses and crisis situations might influence the allocation of OCVs differently. Finally, we describe the effects by African region (West, East, Central, and South), consistent with the WHO’s definition.

## 3. Results

### 3.1. Sample

Twenty-two countries were exposed to 576 outbreaks in total between 2013 and 2019 (3.7 outbreaks per year per country on average, while the dataset does not record outbreaks for Burundi, Sierra Leone, or Sudan). On average, during these outbreaks there were about 519.0 suspected cases, 36.4 confirmed cases, and 4.81 deaths (if data were available). The average outbreak lasted 12.9 weeks, reaching a threshold of 10.1 cases per week, an attack rate of 5.24 per 1000 people, a case fatality rate of 1.85, and a mean instantaneous reproductive number over the first week of an outbreak (R_0_) of 1.90. Most outbreaks occurred in rural areas (82.6%) with high population density (6245.9 people per km^2^).

Over the study period, 18 countries sent 85 requests, 74 were considered, 60 were approved, and those accounted for 96 deliveries. About 88.14 million doses were requested, 73.59 million doses were approved, but only 50.62 million doses were delivered. Table 1, Panel B shows that, in terms of requests, 63.5% of requests were from the MOH, 21.2% from WHO and 11.8% from MSF. In terms of agents approving the requests, IGC approved 85.1% of the requests. On average, it took 299.6 days from the last outbreak to a first request, 10.4 days to make a decision, but 83.3 days to deliver OCVs to the countries. Shanchol was delivered in 46.9% of cases, Euchivol Plus in 36.5% of cases, and Euchivol in the remaining 14.6% of cases. The majority of deliveries were reactive to outbreaks (77.6%) rather than a preventive allocation. Table A4 presents similar summary statistics on cholera outbreaks and OCV at the month–country level.

**Figure 2:**
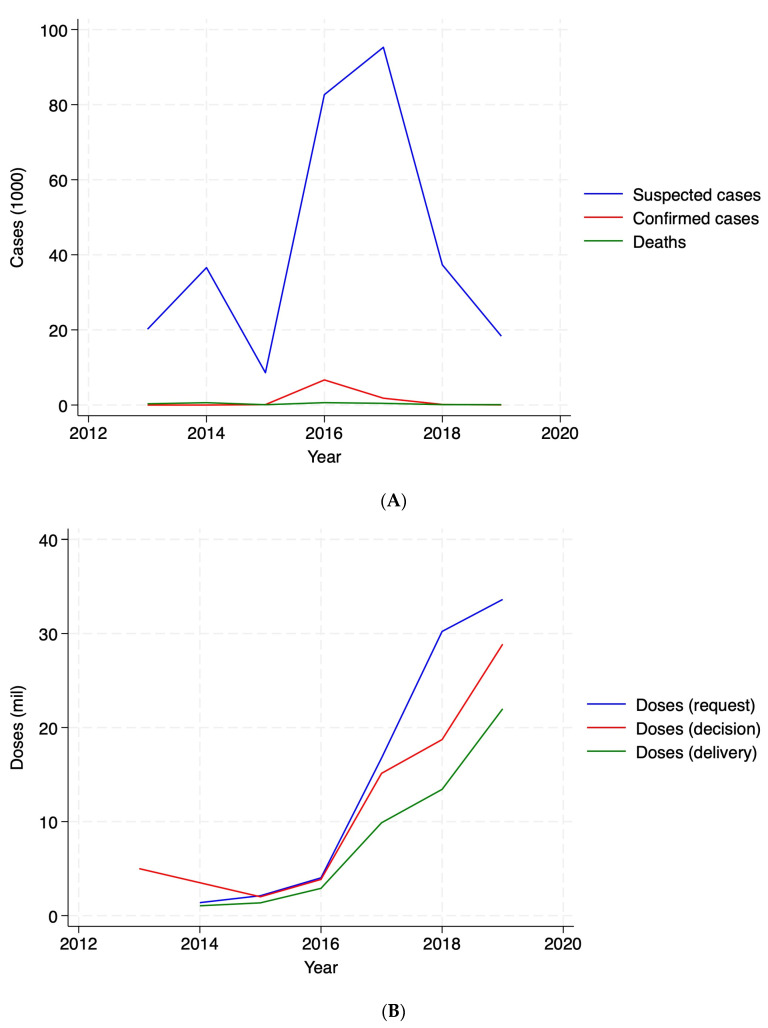
Cholera cases and vaccine doses. Panel (**A**): cases (1000). Panel (**B**): vaccine doses (million).

**Figure A1:**
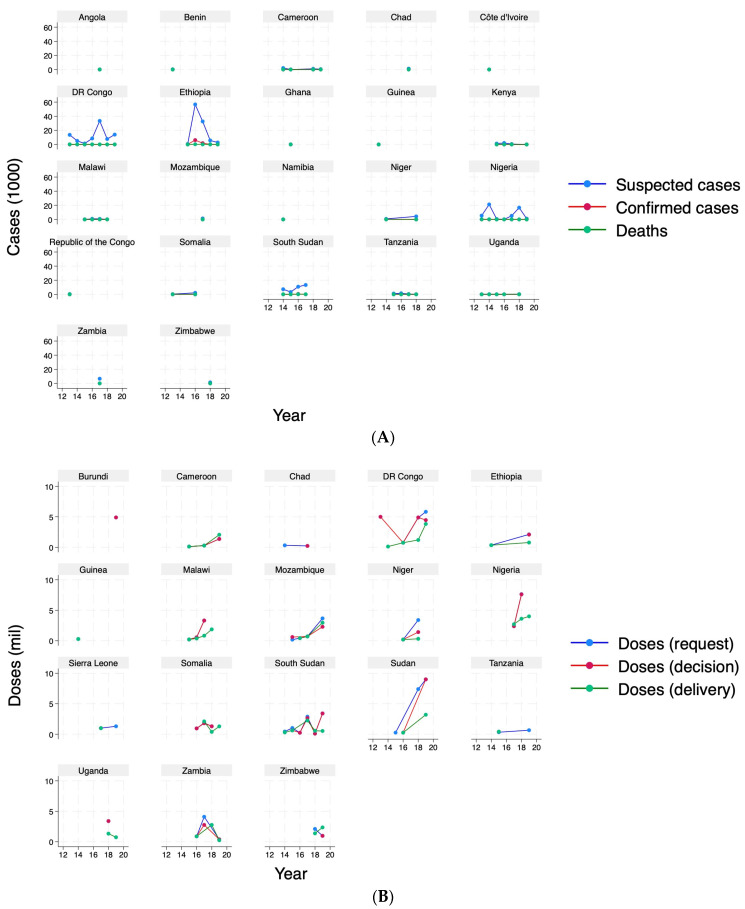
Cholera cases and vaccine doses, by country. Panel (**A**): cases (1000). Panel (**B**): vaccine doses (million).

**Figure A2:**
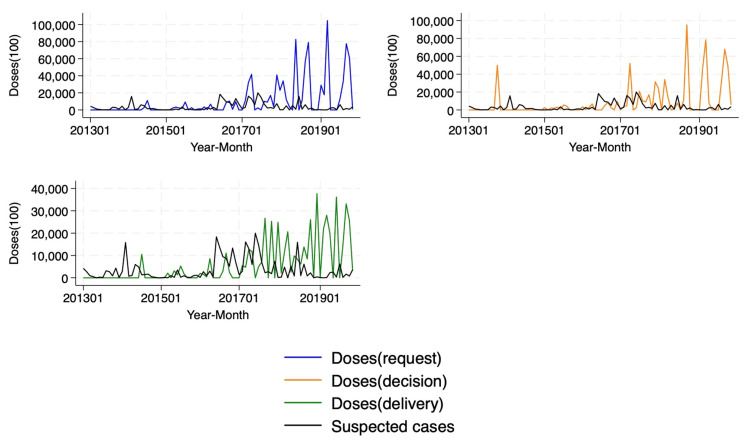
Cholera (suspected) cases and vaccine doses (1000).

In terms of cases, cholera outbreaks showed a peak in 2017 (Figure 2, Panel A), as driven by Ethiopia and Democratic Republic of the Congo (Figure A1, Panel B). In terms of OCVs, Figure 2, Panel B depicts total doses (million) over the study period, suggesting that deliveries are not matching either requests or decisions. Figure A1, Panel B in Appendix A shows variation across countries, suggesting that some countries had never received a dose despite requesting, such as Burundi and Chad, and some received vaccines without a request, such as Cameroon, Democratic Republic of the Congo, Malawi, Nigeria, Somalia, and South Sudan as measured, over the study period. Figure A2 provides a comprehensive visual representation of patterns over time. It simultaneously depicts suspected cholera cases—the primary measure used to define a cholera outbreak in the analysis—and vaccine doses by requests, decisions, and deliveries (in 1000s) at the year–month level, highlighting considerable variability over time.

### 3.2. Allocation of Cholera Vaccines

Table 2 presents the impact of cholera outbreaks on the allocation of OCVs. We find that countries experiencing a cholera outbreak are 31.7 percentage points more likely to request vaccines and 36.5 percentage points more likely to receive a vaccine delivery in the same month as the outbreak. As expected, deliveries were also more likely one month and three months after the outbreak, increasing by 47.4 and 32 percentage points, respectively. The number of doses delivered was also significantly higher—by 245,565 doses—one month after an outbreak. These results remain robust when using the mean R0 reproduction number of the outbreak as the main regressor, though of a smaller magnitude (Table A5).

Requests were mainly made by AMP, while decisions were made by ICG. In the same month as the outbreak, the delivery of the Shanchol vaccine was more likely. One month later, Euvichol was also delivered, and three months after the outbreak, deliveries of Euvichol Plus were more common. Finally, the analysis also confirms that after outbreaks, the probability of obtaining vaccines through a reactive mechanism was 48.4 percentage points higher compared to preventive allocation (Table A6).

When examining the number of events (requests, decisions, and deliveries) and the time elapsed from the outbreak to requests, from requests to decisions, and from decisions to deliveries (Table 3), we find results consistent with those in Table 2. Countries that experienced a cholera outbreak had 0.368 more requests and 0.402 more deliveries in the same month as the outbreak. They also had 0.338 more decisions and 0.532 more deliveries one month after the outbreak. However, we do not find statistically significant results three months after the outbreak. In the IV empirical specification, we also find no evidence that outbreaks impact the time taken by institutions to complete each event. Note that in the OLS specification, we find that countries experiencing a cholera outbreak requested OCVs 28.283 days earlier than countries without an outbreak. This is not surprising, but the large magnitude of the coefficient is primarily driven by the small number of observations where we could link a request to a previous outbreak and thus construct the average number of days from a previous outbreak to a request at the month level.

### 3.3. Heterogeneous Allocation of Cholera Vaccines

When we explore the heterogeneity of the impacts of cholera outbreaks on OCV allocation one month after the outbreak across various dimensions of vulnerability, we find that vaccines are more likely to be requested, allocated, and delivered in countries defined with strong institutions and those not facing crisis situations. When analyzing effects by African region, we find that vaccines are more likely to be allocated in the central (and southern) parts of the continent rather than in East and West Africa. This may be driven by the Democratic Republic of the Congo, which experienced the majority of outbreaks (N = 56) over the study period (Table 4).

## 4. Discussion

In this paper, we examine the allocation of oral cholera vaccines (OCVs) across 25 African countries, between 2013 and 2019. We address the endogeneity of cholera outbreaks by construing an “index of cholera risk” at the country–month level using machine learning algorithms, such as the least absolute shrinkage and selection operator (LASSO) estimation. By assessing the relative importance of socio-demographic, governance, and weather variables in predicting cholera outbreaks, we use the index of cholera risk as an instrumental variable to predict the likelihood of suspected cases and thus to estimate the impact of cholera outbreaks on OCV allocation, including requests, decisions, and deliveries; days to each event; agents involved; and type of mechanism (reactive or preventive).

We find that, during the study period, the majority of OCVs (77.4%) were allocated reactively. Following an outbreak, governments took an average of 299.6 days to request doses, international agencies took 10.4 days to decide on these requests, and it took 84 days for vaccines to be delivered. Countries experiencing a cholera outbreak were 31.7 and 36.5 percentage points more likely to request and receive a vaccine delivery in the same month as the outbreak, respectively. We confirmed that the probability of obtaining vaccines through a reactive mechanism was 48.4 percentage points higher compared to preventive allocation.

Moreover, we identify important heterogeneities in vaccine distribution: countries with stronger institutions and those not experiencing crisis situations were more likely to request, receive, and successfully deploy vaccines, raising concerns about equitable access in more fragile settings. Regional disparities further emphasize these challenges, as Central Africa receives a larger share of vaccine allocations, likely driven by recurrent outbreaks in the Democratic Republic of the Congo. These findings underscore the need for proactive and targeted strategies to ensure timely and equitable vaccine distribution, particularly in vulnerable and crisis-affected regions where institutional capacity may be weaker.

We contribute to the current limited literature on OCV allocation, which has largely been confined to modeling studies [14,15], by quantitatively examining the allocation of cholera vaccines across Africa. While previous work has offered valuable insights into theoretical allocation frameworks, our study provides a comprehensive, empirical analysis of OCV distribution patterns in real-world settings. By using a machine learning-based cholera risk index and addressing key endogeneity concerns, our study advances the literature by offering a more nuanced understanding of the allocation process. Our findings highlight substantial delays in vaccine allocation, especially in reactive contexts, and underscore the disparities in vaccine access between more and less vulnerable countries. These insights are crucial for informing policies aimed at optimizing vaccine distribution, improving response times, and ensuring that the most vulnerable populations in fragile settings are not left behind. Additionally, we shed light on the importance of governance and institutional capacity in determining vaccine allocation, an aspect overlooked in previous studies.

Given the resurgence of cholera in many regions, particularly in sub-Saharan Africa, understanding how vaccines should be allocated remains crucial for effective public health responses. The world is facing significant shortages of OCVs, not only due to the increasing number of cholera outbreaks but also because of vaccine manufacturers’ limited interest in production, driven by low economic returns [35,36]. However, recent efforts by companies in South Korea, India, and South Africa to increase production capacity for simplified OCV formulations and ramp-up manufacturing following WHO prequalification over the next few years [35] present a promising development. Despite these efforts, the dual challenge of rising demand and constrained supply necessitates a closer examination of the factors influencing OCV distribution to ensure equitable access, particularly in high-risk regions. Our study highlights the need for proactive planning and stronger governance mechanisms to ensure equitable and efficient OCV allocation across Africa, reinforcing the importance of preparing not only for preventive but also reactive strategies to combat cholera effectively.

Our study is not without limitations. First, the accuracy and completeness of cholera case reporting vary across countries, particularly in regions with weak surveillance systems. Underreporting or misclassification of cases could affect the estimated cholera risk index and the analysis of vaccine allocation. Our primary analysis relies on suspected cholera cases as the main variable of interest, as confirmed cases and death records have missing values. Second, while we address endogeneity concerns with an instrumental variable approach, this strategy may not fully eliminate all sources of bias. Unobserved factors, such as political will or logistical constraints, may still influence both cholera outbreaks and OCV allocation. Third, distinguishing between reactive and preventive vaccine allocation is challenging due to the recurring nature of cholera outbreaks and the continuous distribution of OCVs. Some preventive campaigns may be indirectly influenced by recent outbreaks, complicating the identification of clear causal links. Additionally, the short interval between cholera outbreaks (about 3 months) makes it difficult to conduct a robust event-study analysis, as the frequent recurrence of outbreaks results in overlap between the post-event period of one outbreak and the pre-event period of the next one. Furthermore, the long delays in vaccine requests and deliveries mean that responses to an outbreak often extend into subsequent outbreak periods. Future research with a larger sample size could explore alternative causal inference methods to better assess the impact of outbreaks on OCV allocation. Finally, our analysis focuses on the country level within Africa, where governance capacity, health system infrastructure, and international agency engagement vary widely. The geographic scope of our analysis also limits the generalizability of our findings to regions outside Africa. Similarly, our study covers the period from 2013 to 2019, using cholera outbreak data compiled by the GTFCC. More recent developments in vaccine stockpiles, funding, and distribution mechanisms may have influenced allocation dynamics in subsequent years. Future research, particularly with more granular and recent data, could examine vaccine allocation beyond this period and assess OCV deployment at subnational levels to better understand regional and community-level disparities.

Our findings carry several important policy implications for improving the timeliness, equity, and effectiveness of OCV allocation. First, delays in vaccine requests highlight the need to streamline and simplify the application and approval processes. Establishing fast-track mechanisms—such as pre-approved outbreak response protocols or automated triggers based on early surveillance data—could reduce the time lag observed between outbreak onset and vaccine request. Second, while vaccination plays a critical role, it must be part of an integrated cholera control strategy. Investments in strengthening health systems, expanding access to safe water and sanitation, and improving surveillance infrastructure remain essential to reduce both the incidence of cholera and reliance on emergency vaccination campaigns. Finally, the proposed cholera risk index offers a promising, data-driven approach to guide vaccine deployment. While further validation is needed, its use as a decision-support tool could help prioritize high-risk areas and ensure more proactive, needs-based allocation—provided that it remains accessible, interpretable, and operationally feasible for use by national health authorities and global partners alike.

## 5. Conclusions

Our findings highlight the reactive nature of OCV allocation, with vaccine requests and deliveries significantly increasing following outbreaks. However, despite this responsiveness, delays in vaccine deployment and discrepancies between requests, approvals, and deliveries suggest inefficiencies in the allocation process. Strengthening institutional capacity and ensuring equitable vaccine distribution, particularly in crisis-affected regions, remain crucial for improving cholera prevention and response efforts. Future research should examine the drivers of these delays and assess more proactive allocation strategies, especially in high-risk, low-capacity contexts, to inform fairer and more efficient vaccine delivery systems.

## Figures and Tables

**Table 1:** Characteristics of cholera outbreaks and vaccines.

	Mean	SD	Min	Max	N Obs
Panel A. Cholera outbreaks					
Tot suspected cases	519.0	1189.4	5	15,871	576
Tot confirmed cases	36.4	212.3	0	2751	242
Total deaths	4.8	9.5	0	75	487
Duration (weeks)	12.9	7.1	3	48	576
Threshold	10.1	77.8	0	1656.9	576
Attack rate	5.2	28.9	0	465.1	576
Case fatality rate	1.8	2.9	0	21.6	487
Mean R0	1.9	0.34	1.1	3.39	576
Population (area outbreak)	9015.0	20,930.2	0.1	200,479	576
Population density	6245.9	120,844.3	2.4	2,900,000	576
Rural	82.6	37.9	0	100	576
Pop less than 10,000	1.2	11.0	0	100	576
Pop between 10,000 and 100,000	20.3	40.3	0	100	576
Pop between 100,000 and 1,000,000	63.2	48.3	0	100	576
Pop more than 1,000,000	15.3	36.0	0	100	576
WHO AFRO region	99.3	8.31	0	100	576
Panel B. Cholera vaccines					
Total doses	832,726.3	1,132,318.9	40,000	7,400,000	255
Any request	33.3	47.2	0	100	255
Any decision	29.0	45.5	0	100	255
Any delivery	37.6	48.5	0	100	255
Request approved	81.1	39.4	0	100	74
Request agent MOH	63.5	48.4	0	100	85
Request agent WHO	21.2	41.1	0	100	85
Request agent AMP	2.35	15.2	0	100	85
Request agent MSF	11.8	32.4	0	100	85
Request agent STC	1.18	10.8	0	100	85
Approved agent GTFCC	14.9	35.8	0	100	74
Approved agent ICG	85.1	35.8	0	100	74
Duration request (days)	267.8	482.9	0	1697	68
Duration decision (days)	10.4	23.2	0	180	73
Duration delivery (days)	83.3	126.7	4	590	83
Vaccine Euvichol	14.6	35.5	0	100	96
Vaccine Euvichol Plus	36.5	48.4	0	100	96
Vaccine Shanchol	46.9	50.2	0	100	96
Mechanism—Preventive	22.4	41.7	0	100	255
Mechanism—Reactive	77.6	41.7	0	100	255

Notes: The table presents summary statistics on cholera outbreaks (Panel A) and vaccines (Panel B). Means (in % for binary variables) are reported along with standard deviations, minimum and maximum, and number of observations in the dataset.

**Table 2:** The impact of cholera outbreaks on allocation of vaccines.

	(1)	(2)	(3)	(4)	(5)	(6)
	Requests		Decisions		Deliveries	
	Any	Number of doses	Any	Number of doses	Any	Number of doses
Panel A: Time T						
Any outbreak (OLS)	−0.005	58,555.463 *	0.009	25,124.194	0.014	2887.914
	(0.016)	(31,117.942)	(0.015)	(27,487.831)	(0.017)	(13,142.015)
Any outbreak (IV)	0.317 *	222,007.120	0.176	200,055.388	0.365 **	115,103.415
	(0.164)	(300,933.855)	(0.145)	(266,681.698)	(0.177)	(128,520.329)
Observations	2100	2100	2100	2100	2100	2100
Mean Dep. Var.	0.0338	41971	0.0300	35041	0.0410	24104
Panel B: Time T + 1						
Any outbreak (OLS)	0.027 *	15,631.338	0.012	16,799.153	0.041 **	26,160.278 **
	(0.016)	(31,148.978)	(0.015)	(27,475.541)	(0.017)	(13,156.631)
Any outbreak (IV)	0.217	113,468.394	0.245	221,985.055	0.474 **	245,564.992 *
	(0.154)	(299,855.860)	(0.149)	(267,529.976)	(0.185)	(134,927.105)
Observations	2099	2099	2099	2099	2099	2099
Mean Dep. Var.	0.0338	41991	0.0300	35058	0.0410	24115
Panel C: Time T + 3						
Any outbreak (OLS)	−0.016	24,530.069	−0.002	37,168.555	0.039 **	13,706.584
	(0.015)	(31,148.397)	(0.015)	(27,491.764)	(0.017)	(13,174.671)
Any outbreak (IV)	−0.124	−152,266.581	−0.089	63,999.967	0.320 *	213,713.445
	(0.151)	(301,813.116)	(0.142)	(264,300.439)	(0.171)	(133,809.120)
Observations	2097	2097	2097	2097	2097	2097
Mean Dep. Var.	0.0339	42,031	0.0300	35,092	0.0410	24,138

Notes: The table presents the main estimates for requests, decisions, and deliveries of cholera vaccines using ordinary least squares (OLS) in Equation (1) and instrumental variable (IV) in Equation (3). The number of observations and the mean of the dependent variable are reported. Observations are at the country–month level from 2013 to 2019. Panel A describes the effects in the same month as a cholera outbreak (time t), Panel B describes the effects one month later, and Panel C describes the effects three months after a cholera outbreak. Standard errors are reported in parentheses. *** *p* < 0.01, ** *p* < 0.05, * *p* < 0.1.

**Table 3:** The impact of cholera outbreaks on additional number of events and days to events.

	(1)	(2)	(3)	(4)	(5)	(6)
	Requests		Decisions		Deliveries	
	Events	Days to	Events	Days to	Events	Days to
Panel A: Time T						
Any outbreak (OLS)	−0.015	−28.283 ***	0.010	0.223	0.023	−3.068
	(0.020)	(8.066)	(0.018)	(0.409)	(0.020)	(2.481)
Any outbreak (IV)	0.368 *	−66.152	0.283	−0.633	0.402 *	14.376
	(0.208)	(77.893)	(0.185)	(3.929)	(0.206)	(24.428)
Observations	2100	2100	2100	2100	2100	2099
Mean Dep. Var.	0.0405	7.520	0.0352	0.331	0.0457	3.105
Panel B: Time T + 1						
Any outbreak (OLS)	0.027 *	15,631.338	0.012	16,799.153	0.041 **	26,160.278 **
	(0.016)	(31,148.978)	(0.015)	(27,475.541)	(0.017)	(13,156.631)
Any outbreak (IV)	0.236	15.809	0.338 *	6.582	0.532 **	29.682
	(0.195)	(77.840)	(0.188)	(4.164)	(0.216)	(24.415)
Observations	2099	2099	2099	2099	2099	2098
Mean Dep. Var.	0.0405	7.523	0.0353	0.331	0.0457	3.106
Panel C: Time T + 3						
Any outbreak (OLS)	−0.016	24,530.069	−0.002	37,168.555	0.039 **	13,706.584
	(0.015)	(31,148.397)	(0.015)	(27,491.764)	(0.017)	(13,174.671)
Any outbreak (IV)	−0.120	79.890	−0.003	−3.052	0.325	32.692
	(0.192)	(79.700)	(0.176)	(4.005)	(0.199)	(24.480)
Observations	2097	2097	2097	2097	2097	2096
Mean Dep. Var.	0.0405	7.531	0.0353	0.331	0.0458	3.109

Notes: The table presents the main estimates for number of, and days to requests, decisions, and deliveries of cholera vaccines using ordinary least squares (OLS) in Equation (1) and instrumental variable (IV) in Equation (3). The number of observations and the mean of the dependent variable are reported. Observations are at the country–month level from 2013 to 2019. Panel A describes the effects in the same month as a cholera outbreak (time t), Panel B describes the effects one month later, and Panel C describes the effects three months after a cholera outbreak. Standard errors are reported in parentheses. *** *p* < 0.01, ** *p* < 0.05, * *p* < 0.1.

**Table 4:** Heterogeneous impacts of cholera outbreaks on allocation of vaccines.

	(1)	(2)	(3)	(4)	(5)	(6)
At time T + 1:	Requests		Decisions		Deliveries	
Any outbreak (IV), by:	Any	Number of doses	Any	Number of doses	Any	Number of doses
Panel A. Institutions						
Weak institutions	0.147	−134,808.894	0.224	137,230.919	0.351	32,157.132
	(0.295)	(705,048.979)	(0.288)	(637,126.970)	(0.329)	(283,872.043)
Observations	899	899	899	899	899	899
Strong institutions	0.256 *	267,162.377	0.244 *	217,037.552	0.454 **	267,640.320 **
	(0.153)	(207,731.472)	(0.146)	(169,606.005)	(0.184)	(113,104.641)
	1200	1200	1200	1200	1200	1200
Panel B. Crisis						
Crisis	−0.089	−368,176.662	0.044	−145,792.791	0.037	−156,956.988
	(0.192)	(462,040.563)	(0.184)	(409,032.092)	(0.205)	(188,573.994)
Observations	701	701	701	701	701	701
No Crisis	0.467 *	481,355.048	0.433 *	391,095.705	0.804 **	521,356.897 **
	(0.259)	(333,583.648)	(0.243)	(273,423.953)	(0.333)	(215,681.618)
Observations	1396	1396	1396	1396	1396	1396
Panel C. Africa Region						
West and East	0.346	1,043,485.56	0.649	1,346,289.88	0.219	398,336.826
	(0.568)	(1,384,256.64)	(0.730)	(1,547,463.60)	(0.515)	(596,624.072)
Observations	1008	1008	1008	1008	1008	1008
Central and South	0.095	−323,216.610	0.125	−162,324.153	0.539 **	221,961.969
	(0.179)	(367,952.662)	(0.175)	(307,006.411)	(0.219)	(147,556.719)
Observations	1091	1091	1091	1091	1091	1091
Mean Dep. Var.	0.0338	41,991	0.0300	35,058	0.0410	24,115

Notes: The table presents the main estimates for requests, decisions, and deliveries of cholera vaccines using ordinary least squares (OLS) in Equation (1) and instrumental variable (IV) in Equation (3), by weak or strong institutions (Panel A), by crisis or no crisis situation (Panel B), and by WHO AFRO regions. Weak institutions are defined as those scoring below the median for government effectiveness and control of corruption; countries are classified with a crisis if they have refugee populations and number of conflicts above the median. We use the WHO definition of regions in Africa to define countries in east and west versus central and south parts of the continent. The number of observations and the mean of the dependent variable are reported. Observations are at the country–month level from 2013 to 2019. Panel A describes the effects in the same month as a cholera outbreak (time t), Panel B describes the effects one month later, and Panel C describes the effects three months after a cholera outbreak. Standard errors are reported in parentheses. *** *p* < 0.01, ** *p* < 0.05, * *p* < 0.1.

## Data Availability

The data used for analysis are publicly available.

## Abbreviations

The following abbreviations are used in this manuscript:

AMP:	Agence de Médecine Préventive
DALYs:	Disability-Adjusted Life Years
GAVI:	Gavi, the Vaccine Alliance
GTFCC:	Global Task Force on Cholera Control
ICG:	International Coordinating Group
IFRC:	International Federation of Red Cross and Red Crescent Societies
IV:	instrumental variable
LASSO:	least absolute shrinkage and selection operator
MSF:	Médecins Sans Frontières
OCV:	oral cholera vaccine
OLS:	ordinary least squares
STC:	STChealth
WASH:	water, sanitation, and hygiene
WHO:	World Health Organization

## Appendix A

**Table A1:** List of countries in the sample.

Country Name	With Outbreak	With Vaccine Request
Angola	X	
Benin	X	
Burundi		X
Cameroon	X	X
Chad	X	X
Côte d’Ivoire	X	
Democratic Republic of the Congo	X	X
Ethiopia	X	X
Ghana	X	
Guinea	X	X
Kenya	X	
Malawi	X	X
Mozambique	X	X
Namibia	X	
Niger	X	X
Nigeria	X	X
Republic of the Congo	X	
Sierra Leone		X
Somalia	X	X
South Sudan	X	X
Sudan		X
Tanzania	X	X
Uganda	X	X
Zambia	X	X
Zimbabwe	X	X

**Table A2:** Description of variables and databases.

Covariates	Definition	Data Source	Available Years
Socio-demographic variables			
GDP per capita	GDP per capita is gross domestic product divided by midyear population, in current U.S. dollars.	The World Bank	1960–2022
Population density	People per sq. km of land area is mid-year population divided by land area in square kilometers.	The World Bank	1961–2021
Urban population	Share of the population living in urban areas.	The World Bank	1960–2023
Unimproved drinking water	Share of the population using unimproved water sources as defined by the World Health Organization.	WHO/UNICEF Joint Monitoring Programme	2000–2022
Unimproved sanitation services	Share of the population using unimproved sanitation services. Sanitation services refer to the management of excreta from the facilities used by individuals. Unimproved sanitation for example refers to use of pit latrines without a slab or platform, hanging latrines or bucket latrines.	WHO/UNICEF Joint Monitoring Programme	2000–2022
Refugees’ population	Refugee population by country or territory of origin	The World Bank	1960–2023
Net migration	Net total of migrants during the period, that is, the number of immigrants minus the number of emigrants, including both citizens and noncitizens.	The World Bank	1960–2023
Total number of conflicts	The national conflict event by country and month. We reconstructed the monthly total number of conflicts.	Armed Conflict Location and Event Data (ACLED)	1997–2024
Total number of fatalities due to conflict	The national fatalities due to conflict events by country and month. We reconstructed the monthly total number of fatalities due to conflict.	Armed Conflict Location and Event Data (ACLED)	1997–2024
Governance variables			
Election	Number of elections in a given year	Adam Carr’s Election Archive: Psephos	2013–2019
Control of corruption	Estimated score on “Control of corruption”, in units of a standard normal distribution, and measures the extent to which public power is exercised for private gain.	The World Bank, Worldwide Governance Indicators	1996–2023
Government effectiveness	Estimated score on “Government effectiveness”, in units of a standard normal distribution, and captures perceptions of the quality of policy formulation and implementation, and the credibility of the government’s commitment to policies.	The World Bank, Worldwide Governance Indicators	1996–2023
Political stability and absence of violence/terrorism	Estimated score on “Political stability and absence of violence/terrorism”, in units of a standard normal distribution and captures the likelihood of political instability and/or politically motivated violence.	The World Bank, Worldwide Governance Indicators	1996–2023
Weather variables			
Average mean surface air temperature	Monthly average mean surface air temperature in degrees Celsius. We constructed the average mean surface air temperature between 20 and 25 degrees Celsius.	The World Bank, Climate Change Knowledge Portal	1901–2022
Average accumulated precipitation	Monthly average accumulated precipitation in millimeters.	The World Bank, Climate Change Knowledge Portal	1901–2022
Number of floods	Number of floods in a given month.	Global Flood Database	2000–2018

**Table A3:** First stage estimation, by measure of cholera outbreak.

	(1)	(2)	(3)	(4)	(5)	(6)	(7)	(8)	(9)	(10)	(11)	(12)	(13)
	Any Outbreak			Tot Number of Cases			Threshold	Duration	Time to Peak	Attack Rate	CFR	R0	Cumulative
	Suspected	Confirmed	Deaths	Suspected	Confirmed	Deaths	(Mean)	(Mean)	(Mean)	(Mean)	(Mean)	(Mean)	Cases
Cholera Risk Index	0.024 **	0.005	0.013 **	16.136	0.784	0.213	0.042	0.209 **	0.093 **	−0.004	0.077 **	0.045 **	29.646
	(0.009)	(0.003)	(0.006)	(12.309)	(0.866)	(0.158)	(0.045)	(0.097)	(0.040)	(0.025)	(0.032)	(0.017)	(20.393)
Observations	2100	2100	2100	2100	2100	2100	2100	2100	2100	2100	2100	2100	2100
Mean Dep. Var.	0.100	0.0190	0.0700	142.4	4.196	1.115	0.852	1.259	0.478	0.382	0.217	0.186	223.5

Notes: The table presents the main estimates for the first stage IV estimation (equation (2)) across different outbreak characteristics. The empirical model is an ordinary least squares (OLS). The number of observations and the mean of the dependent variable are reported. Observations are at the country–month level from 2013 to 2019. Standard errors are reported in parentheses. *** *p* < 0.01, ** *p* < 0.05, * *p* < 0.1.

**Table A4:** Characteristics of cholera outbreaks and vaccines (month-level dataset).

	Mean	SD	Min	Max	N Obs
Panel A. Cholera outbreaks					
Tot suspected cases	142.4	964	0	19157	2100
Tot confirmed cases	4.2	128.2	0	5627	2100
Total deaths	1.1	6.7	0	106	2100
Duration (weeks)	1.3	4.3	0	30	2100
Threshold	0.8	12.7	0	419.8	2100
Attack rate	0.4	3.6	0	89.2	2100
Case fatality rate	0.2	1.3	0	21.6	2100
Mean R0	0.2	0.6	0	2.74	2100
Panel B. Cholera vaccines					
Any request	3.4	18.1	0	100	2100
Any decision	3	17.1	0	100	2100
Any delivery	4.1	19.8	0	100	2100
Total doses—request	41,971.3	359,406.7	0	7,400,000	2100
Total doses—decisions	35,041.4	316,824.4	0	5,700,000	2100
Total doses—delivery	24,104	154,747.6	0	2,700,000	2100
Number of requests	0.04	0.2	0	3	2100
Number of decisions	0	0.2	0	3	2100
Number of deliveries	0	0.2	0	3	2100
Request approved	2.5	15.7	0	100	2100
Request agent MOH	2.2	14.6	0	100	2100
Request agent WHO	0.6	7.8	0	100	2100
Request agent AMP	0.1	3.1	0	100	2100
Request agent MSF	0.5	6.9	0	100	2100
Request agent STC	0	2.2	0	100	2100
Approved agent GTFCC	0.5	6.9	0	100	2100
Approved agent ICG	2.6	15.8	0	100	2100
Approved agent Loan	0	0	0	0	2100
Duration decision (days)	0.3	4.6	0	180	2100
Duration delivery (days)	3.1	28.9	0	590	2099
Vaccine Euvichol	0.6	7.85	0	100	2100
Vaccine Euvichol Plus	1.6	12.4	0	100	2100
Vaccine Shanchol	1.9	13.7	0	100	2100
Mechanism—Preventive	2.4	15.2	0	100	2100
Mechanism—Reactive	4.6	20.9	0	100	2100

Notes: The table presents summary statistics on cholera outbreaks (Panel A) and vaccines (Panel B). Means (in % for binary variables) are reported along with standard deviations, minimum and maximum, and number of observations in the dataset.

**Table A5:** The impact of cholera outbreaks on allocation of vaccines (robustness check with mean R0 as regressor).

	(1)	(2)	(3)	(4)	(5)	(6)
	Requests		Decisions		Deliveries	
	Any	Number of Doses	Any	Number of Doses	Any	Number of Doses
Panel A: Time T						
Mean R0 (OLS)	−0.005	22,384.242	0.001	4991.843	0.007	−460.434
	(0.008)	(16,424.032)	(0.008)	(14,504.527)	(0.009)	(6933.479)
Mean R0 (IV)	0.164 *	115,034.849	0.091	103,660.376	0.189 **	59,641.799
	(0.085)	(156,162.074)	(0.075)	(138,405.873)	(0.091)	(66,630.837)
Observations	2100	2100	2100	2100	2100	2100
Mean Dep. Var.	0.0338	41971	0.0300	35,041	0.0410	24,104
Panel B: Time T + 1						
Mean R0 (OLS)	0.017 **	5030.271	0.007	7266.510	0.021 **	11,663.578 *
	(0.008)	(16,434.088)	(0.008)	(14,495.866)	(0.009)	(6943.096)
Mean R0 (IV)	0.112	58,794.736	0.127 *	115,023.685	0.245 **	127,241.855 *
	(0.080)	(155,412.121)	(0.077)	(138,610.096)	(0.096)	(69,918.320)
Observations	2099	2099	2099	2099	2099	2099
Mean Dep. Var.	0.0338	41,991	0.0300	35,058	0.0410	24,115
Panel C: Time T + 3						
Mean R0 (OLS)	−0.010	6108.176	−0.001	17,502.306	0.020 **	6297.636
	(0.008)	(16,434.996)	(0.008)	(14,505.222)	(0.009)	(6951.034)
Mean R0 (IV)	−0.064	−78,868.709	−0.046	33,149.722	0.166 *	110,696.014
	(0.078)	(156,134.035)	(0.073)	(136,918.474)	(0.089)	(69,236.070)
Observations	2097	2097	2097	2097	2097	2097
Mean Dep. Var.	0.0339	42,031	0.0300	35,092	0.0410	24,138

Notes: The table presents the main estimates for number of, and days to requests, decisions, and deliveries of cholera vaccines using ordinary least squares (OLS) in Equation (1) and instrumental variable (IV) in Equation (3). The mean R0 number is used as a regressor. The number of observations and the mean of the dependent variable are reported. Observations are at the country–month level from 2013 to 2019. Panel A describes the effects in the same month as a cholera outbreak (time t), Panel B describes the effects one month later, and Panel C describes the effects three months after a cholera outbreak. Standard errors are reported in parentheses. *** *p* < 0.01, ** *p* < 0.05, * *p* < 0.1.

**Table A6:** The impact of cholera outbreaks, by agents and type of vaccines or response.

	(1)	(2)	(3)	(4)	(5)	(6)	(7)	(8)	(9)	(10)	(11)
	Agent for Requests					Agent for Approval		Vaccine			Response
	MOH	WHO	AMP	MSF	STC	GTFCC	ICG	Euvichol	Euvichol Plus	Shanchol	Reactive
Panel A: Time T											
Any outbreak (OLS)	−0.001	−0.005	0.013 ***	−0.013 **	−0.001	0.006	0.001	0.001	0.007	0.009	0.004
	(0.013)	(0.007)	(0.003)	(0.006)	(0.002)	(0.006)	(0.014)	(0.007)	(0.010)	(0.012)	(0.018)
Any outbreak (IV)	0.153	0.075	0.074 **	0.039	−0.003	0.026	0.165	0.027	0.094	0.200 *	0.484 **
	(0.125)	(0.067)	(0.029)	(0.059)	(0.018)	(0.058)	(0.135)	(0.066)	(0.102)	(0.120)	(0.199)
Observations	2100	2100	2100	2100	2100	2100	2100	2100	2100	2100	2100
Mean Dep. Var.	0.0219	0.00619	0.000952	0.00476	0.000476	0.00476	0.0257	0.00619	0.0157	0.0190	0.0457
Panel B: Time T + 1											
Any outbreak (OLS)	0.009	0.011 *	0.006 **	0.009	−0.002	0.006	0.006	0.002	0.013	0.029 **	0.010
	(0.013)	(0.007)	(0.003)	(0.006)	(0.002)	(0.006)	(0.014)	(0.007)	(0.010)	(0.012)	(0.018)
Any outbreak (IV)	0.193	−0.046	0.047 *	0.043	−0.002	0.044	0.232 *	0.128 *	0.139	0.211 *	0.465 **
	(0.127)	(0.066)	(0.027)	(0.058)	(0.018)	(0.058)	(0.139)	(0.071)	(0.104)	(0.119)	(0.196)
Observations	2099	2099	2099	2099	2099	2099	2099	2099	2099	2099	2099
Mean Dep. Var.	0.0219	0.00619	0.000953	0.00476	0.000476	0.00476	0.0257	0.00619	0.0157	0.0191	0.0457
Panel C: Time T + 3											
Any outbreak (OLS)	−0.010	0.007	−0.002	−0.011 *	−0.001	0.004	−0.006	0.010	0.013	0.019 *	−0.000
	(0.013)	(0.007)	(0.003)	(0.006)	(0.002)	(0.006)	(0.014)	(0.007)	(0.010)	(0.012)	(0.018)
Any outbreak (IV)	0.043	−0.098	−0.057 **	0.001	−0.011	−0.056	−0.002	0.073	0.184 *	0.039	0.110
	(0.122)	(0.069)	(0.029)	(0.058)	(0.019)	(0.059)	(0.131)	(0.067)	(0.107)	(0.113)	(0.171)
Observations	2097	2097	2097	2097	2097	2097	2097	2097	2097	2097	2097
Mean Dep. Var.	0.0219	0.00620	0.000954	0.00477	0.000477	0.00477	0.0258	0.00620	0.0157	0.0191	0.0458

Notes: The table presents the main estimates by agents of requests (MOH, WHO, AMP, MSF, STC) and approvals (GTFCC, ICG), type of vaccine (Euvichol, Euvichol Plus, Shanchol), or response (reactive compared to preventive), using ordinary least squares (OLS) in Equation (1) and instrumental variable (IV) in Equation (3). The number of observations and the mean of the dependent variable are reported. Observations are at the country–month level from 2013 to 2019. Panel A describes the effects in the same month as a cholera outbreak (time t), Panel B describes the effects one month later, and Panel C describes the effects three months after a cholera outbreak. Standard errors are reported in parentheses. *** *p* < 0.01, ** *p* < 0.05, * *p* < 0.1.
